# The Preparation of Capsaicin-Chitosan Microspheres (CCMS) Enteric Coated Tablets

**DOI:** 10.3390/ijms141224305

**Published:** 2013-12-13

**Authors:** Jian Chen, Gui-Dong Huang, Si-Rong Tan, Jiao Guo, Zheng-Quan Su

**Affiliations:** Key Research Center of Liver Regulation for Hyperlipemia SATCM/Class III Laboratory of Metabolism SATCM, Guangdong TCM Key Laboratory for Metabolic Diseases, Guangdong Pharmaceutical University, Guangzhou 510006, China; E-Mails: yx07228chenjian@126.com (J.C.); hgd2014@163.com (G.-D.H.); tsrcd408@163.com (S.-R.T.)

**Keywords:** capsaicin, microspheres, enteric coated tablets, HPLC

## Abstract

This study aimed to research the preparation and content determination of capsaicin-chitosan microspheres (CCMS) enteric coated tablets. The core tablets were prepared with the method of wet granulation. Nine formulae were designed to determine the optimal formula of the core tablet. Eudragit L100 was used to prepare the CCMS enteric-coated tablets. The effect of enteric coated formulation variables such as content of talc (10%, 25% and 40%), plasticisers (TEC and DBS), dosage of plasticiser (10%, 20% and 30%) and coating weight (2%, 3% and 5%) were evaluated for drug release characteristics. The *in vitro* release was studied using 0.1 N HCl and pH 6.8 phosphate buffer. Enteric coated tablets without ruptures or swelling behaviour over 2 h in 0.1 N HCl indicated that these tablets showed acid resistance. The accumulated release rate in phosphate buffer (pH 6.8) revealed that the prepared tablets were able to sustain drug release into the intestine and a first-order release was obtained for capsaicin. This research is the first report of the preparation and content determination of CCMS enteric coated tablets. The sustained release behavior of enteric coated formulations in pH 6.8 phosphate buffer demonstrated that it would be a potential drug delivery platform for sustained delivery of gastric irritant drugs.

## 1. Introduction

Obesity, the result of energy intake in excess of energy expenditure, is gradually spreading to low- and middle-income countries, such as China, as a result of novel dietary habits [1,2]. Obesity is also increasingly prevalent among young individuals [3–6]. The prevalence of childhood obesity in urban areas of China increased from 1.5% in 1989 to 12.6% in 1997, while the prevalence of overweight children increased from 14.6% to 28.9% in the same period [7]. The China Family Nutrition Health Survey (CHNS) reported that, as of 2009, the proportion of obese men has reached 11.4%, the female has reached 10.1%, and the proportion of male abdominal obesity was 27.8%, and the female was 45.9% [8]. Obese patients potentially suffer from many serious complications, such as diabetes, cancer, sleep apnea, cardiovascular disease, reproductive abnormalities, osteoarthritis and other ailments [9–15].

As we all know, the severe trend of obesity is an inescapable reality. However, chemical medicaments aimed at obesity are rather rare and have serious side effects [16]. People have thus shifted their focus toward traditional Chinese medicine and natural products to identify medications to aid in weight loss. Chitosan (CTS) is a natural product, an *N*-deacetylation product of chitin, which exists widely in the shells of shrimp and crab, insectile carapaces and the cell walls of fungi and some plants [17]. Because of its limited toxicity, flocculation, biocompatibility, biodegradation and mucoadhesive properties, CTS is used extensively in biomedical applications and pharmaceutical formulations [18–20]. Capsaicin (CAP) is also a natural product, extracted from chilli, which can reduce the absorption of fat in the intestine and accelerate the rate of metabolism of fat, thus promoting the burning of fat in the body. These properties have significant effects for weight reduction [21–23]. However, CAP is a strong stomach irritant, which limits its application.

To overcome this problem, microspheres were prepared that loaded capsaicin onto chitosan (CCMS) by combining the ionic crosslinking and spray-drying method for the high yield and narrow distribution in our previous reports [24,25]. This formulation enabled delivery of a reduced dosage of CAP, increasing the curative effect of weight reduction. In the present study, the preparation and content determination of capsaicin-chitosan microsphere (CCMS) enteric coated tablets were studied. The first step in the process was to compress CCMS into tablets, which were then coated with Eudragit L100 by film coating technology. The preparation technology was optimised and the content of the CAP determined. Eudragit L100 is a pH-dependent, stable, inert, safe and non-irritating coating material. It is insoluble in the stomach and is converted in the intestine into the salt of its active carboxylic acid (–COOH) functional group. CCMS enteric coated tablets could enable CAP release in the intestine and reduce stomach irritation, which, in turn, would increase compliance among obese patients.

## 2. Results and Discussion

### 2.1. Formula Optimisation of CCMS Core Tablets

The experimentally determined properties of nine formulae are shown in Table 1. Carboxymethylcellulose sodium and hydroxypropyl methylcellulose (HPMC) were used as the binder in formulae 1 and 2 respectively. The tablets from formula 2 showed laminating, sticking and picking. So, carboxymethylcellulose sodium was found to be a more suitable binder than HPMC. The Chinese Pharmacopoeia provides criteria that conventional tablets disintegrated completely within 15 min, and the shorter disintegration times have a smaller effect on the drug release. In comparison with formulae 1 and 4, it is clear that formula 3 showed the shortest disintegration time. This indicates that high concentrations of carboxymethylcellulose sodium may lead to delayed disintegration. It was therefore determined that 3% carboxymethylcellulose sodium is the optimal concentration. Formulae 3 and 5 gave similar results, as reflected in Table 2. Magnesium stearate and sodium lauryl sulphate (SLS) both played a good role in lubrication. However, SLS is a moderately toxic material and irritating to the skin and eye [26]. Magnesium stearate is widely used as a pharmaceutical excipient and is generally regarded as being nontoxic following oral administration, and not irritating to the skin [27]. So, magnesium stearate was the most suitable lubricant. Among the last four formulae, the disintegrant used in prescriptions 6, 8 and 9 was sodium starch glycolate that showed better disintegrating effects and reduced the disintegration time. However, formula 8 was determined to be optimal of all the formulae since it required a lower dose of sodium starch glycolate. Overall, the hardnesses, disintegration times and appearances of the formula met the quality inspection criteria for tablets listed by the Chinese Pharmacopoeia.

### 2.2. Preparation of CCMS Enteric Coating

#### 2.2.1. Determination of Capsaicin Equilibrium Solubility

**Figure 1.**
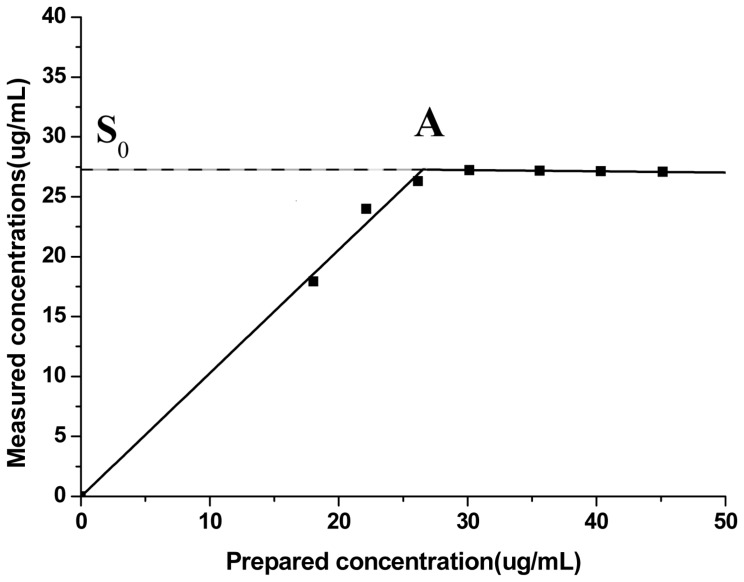
The determination curve of capsaicin equilibrium solubility. Abbreviations: **S_0_**, equilibrium solubility; **A**, the intersection of two lines.

Figure 1 was the determination curve of equilibrium solubility. The intersection A of the two lines (*Y* = 1.0289*X* − 0.0112, *r* = 0.9961. *Y* = −0.0102*X* + 27.5399, *r* = 0.9958) was the equilibrium solubility of capsaicin. The equilibrium solubility of capsaicin was 27.27 μg/mL.

#### 2.2.2. Methodology Experiment

**Figure 2.**
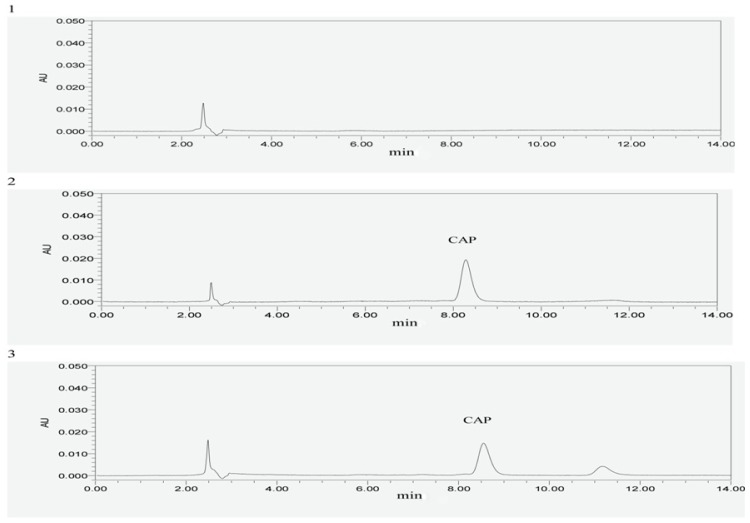
HPLC chromatogram of specificity investigation; **1**: the negative control (an enteric coated tablet without CAP); **2**: standard substance of CAP; **3**: sample of CAP. Abbreviations: CAP, capsaicin.

Linear relationships could be determined from peak area (*Y*) and concentration (*X*): *Y* = 8386.8*X* − 3304.6, *r* = 0.9993. The linear range was 1.81–28.88 μg/mL, which reflected the linear relationship perfectly. CTS, excipients and solvents did not interfere with CAP determination. The relative standard deviations of precision, stability, and recovery experiments were 1.60%, 0.42% and 1.15%, respectively. The average recovery in the recovery experiments was 99.42%. Furthermore, it was revealed from stability experiments that CAP was stable over 24 h. Table 2 shows the results of the recovery experiments. Figure 2 shows the HPLC chromatogram of specificity investigation. The relative standard deviations of precision, stability, and recovery experiments meted with International Conference on Harmonisation of Technical Requirements for Registration of Pharmaceuticals for Human Use (ICH) and Pharmacopoeia Commission of People’s Republic of China (Ch.P.) recommended values.

#### 2.2.3. CAP Dissolution Property from CCMS Core Tablet

**Figure 3.**
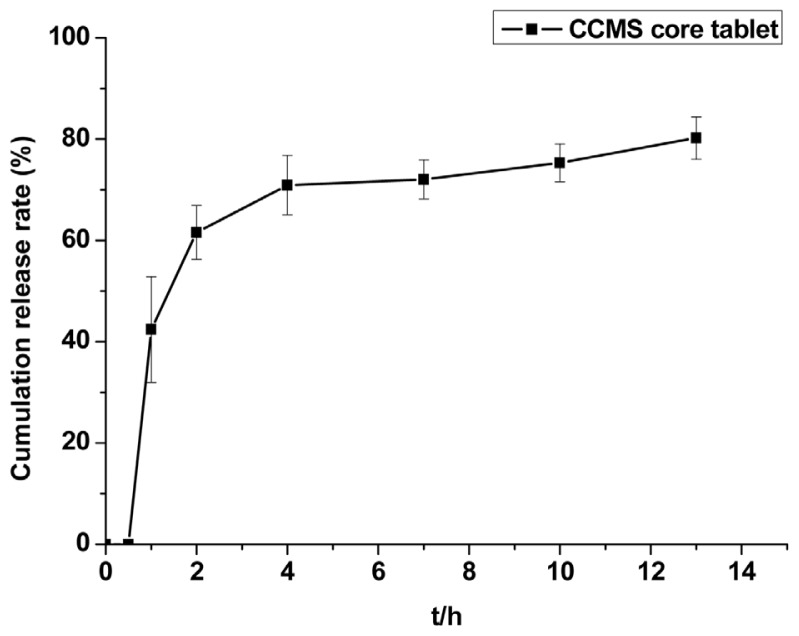
The dissolution property of CAP from the CCMS core tablet. Each point represents the mean ± SD. Abbreviations: CCMS, capsaicin-chitosan microspheres; CAP, capsaicin.

Dissolution properties of the CCMS core tablet were an important consideration for the preparation of CCMS enteric coated tablets. Figure 3 shows the CAP dissolution property from the CCMS core tablet. The core tablets were able to sustain drug release into the pH 6.8 phosphate buffer.

#### 2.2.4. Evaluation of Talc Content

Study of the talc content revealed that 10% talc exhibited a poor anti-sticking effect. Formulations containing 40% talc precipitated easily and blocked the gun, causing difficulties in spraying. The anti-sticking effect of 25% talc was nearly perfect, and at this content did not block the gun, facilitating smooth coating. It was thus determined that the optimum talc content is 25%.

#### 2.2.5. Evaluation of Plasticisers

**Figure 4.**
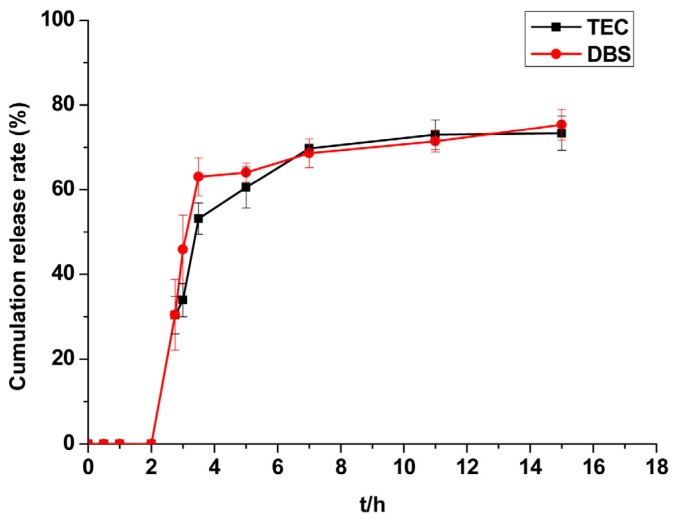
The effect of various plasticisers on the release of CAP from CCMS enteric coated tablets. Each point represents the mean ± SD. Abbreviations: TEC, triethyl citrate; DBS, dibutyl sebacate.

Plasticisers are used to increase the plasticity of highly polymeric materials. They can weaken the Van der Waals forces between polymer chains, increasing mobility and reducing crystallinity. When plasticisers are inserted into polymer chains, the plasticity of the coating material increases and enables even coating. TEC (triethyl citrate) and DBS (dibutyl sebacate) are two common plasticisers used in the preparation of enteric coated tablets. Figure 4 shows the effects of different plasticisers to the release properties of the enteric coated tablets. TEC and DBS had similar effects on the release of CAP. TEC is less expensive than DBS, reducing the cost of industrial production. This makes TEC more suitable as a plasticiser for enteric coated tablets.

#### 2.2.6. Evaluation of TEC Dosage

**Figure 5.**
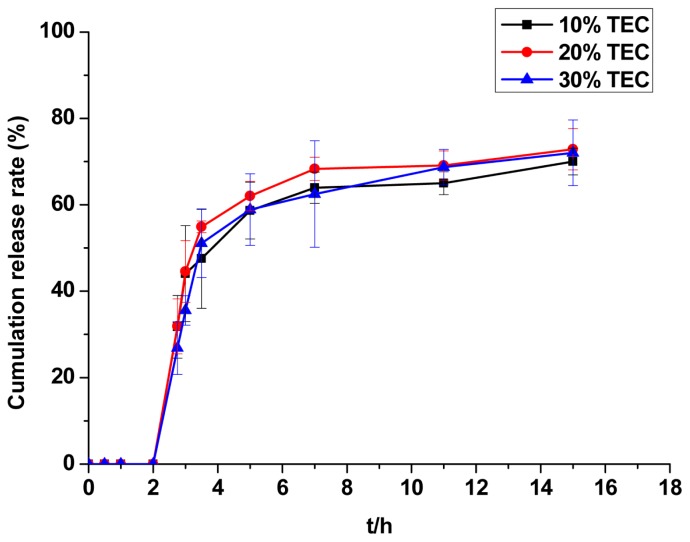
The effect of TEC dosage on the release of CAP from CCMS enteric coated tablets. Each point represents the mean ± SD. Abbreviations: TEC, triethyl citrate.

The results of TEC dosage research are shown in Figure 5. The presence of 30% TEC caused tablets to stick to the coating pan during the coating process. Swelling behaviour and incomplete disintegration phenomena were observed in dissolution experiments, which may have been caused by an improper ratio of coating solution components. When 10% TEC added to the coating solution, swelling behaviour and incomplete disintegration phenomena were also observed in dissolution experiments. The coating process was very successful and the film was rather uniform in the presence of 20% TEC. The release curve of CAP was in accordance with administration regulations for 20% TEC. The optimal TEC content was thus determined to be 20%.

#### 2.2.7. Evaluation of Coating Weight

**Figure 6.**
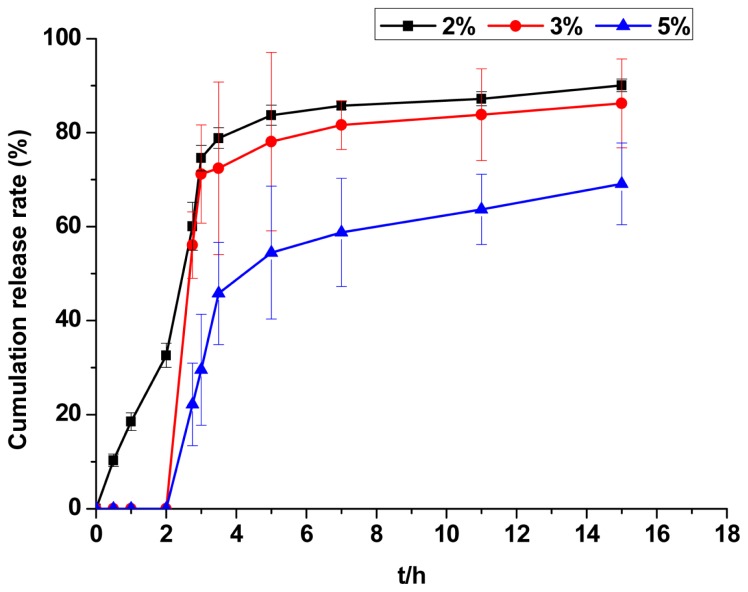
The effect of coating weight on the release of CAP from CCMS enteric coated tablets. Each point represents the mean ± SD.

The drug release behaviours observed with different coating weights in pH 6.8 phosphate buffer solution are shown in Figure 6. It is readily apparent that CAP release was slow and incomplete for the coating weight of 5%. This formulation is unable to release the required therapeutic dose because of the unsuccessful solution of thick film in the intestinal fluid. When the coating weight was 2%, the CCMS enteric coated tablets were not completely coated. These tablets also showed swelling phenomena due to erosion by hydrochloric acid solution. Ultimately, these enteric coated tablets failed altogether. A coating weight of 3% led to a more reasonable drug release curve for CAP. Film formation at this coating weight was also excellent which had no coating film breakage. A coating weight of 3% was therefore determined to be the optimal ratio.

#### 2.2.8. Evaluation of Release Kinetics

The *in vitro* release data obtained from the formulations optimized were fitted to first-order, Higuchi, Riter-Peppas kinetic equation to research the correlation coefficient *R* and mean relative percentage deviation. Table 3 indicated that release of capsaicin from the tablets was a first-order kinetic equation for the highest *R*^2^ value. However, the mean relative percentage deviation of the Riter-Peppas kinetic equation was 5.32%, which was the minimum of all kinetic equations. The Ritger-Peppas equation is a semi-empirical equation integrating diffusion and dissolution. The value of *n* is the release of the index to indicate the drug release mechanism [28]. For the sphere preparations, when *n* is less than 0.43, the drug release mechanism was Fick diffusion. The value of *n* Fitted which was also “a” in the Ritger-Peppas equation was 0.1185, less than 0.43, indicating that the drug release probably was through Fick diffusion. However, chitosan is a type of hydrophilic matrix of materials that is swellable and can form a gel in an aqueous medium. In the process of drug release, the release mechanism is complex, not a simple diffusion mechanism due to the change of skeleton shape, and thickness variation of gel layer and dissolution of materials. So, the release of capsaicin from the tablets was more likely to be a first-order kinetic equation.

### 2.3. Results of Content Determination

The results of content determination experiments on three batches of CCMS enteric coated tablets are shown in the Table 4. The results revealed that each CCMS enteric coated tablet contained, on average, 5.44 mg CAP, and preparation of enteric coated tablets was mature and stable.

## 3. Experimental Section

### 3.1. Chemicals

Chitosan (CTS) was purchased from Shandong AoKang Biotech Ltd. (Shandong, China). The degree of deacetylation (DD) was ≥85%, and the molecular weight was 6.93 × 10^5^ which was determined by viscosity method. The capsaicin (CAP) was purchased from Wuhan Sheng Tian Yu Technology Co., Ltd. (Wuhan, China). The capsaicin standard was purchased from TAUTO BIOTECH Co., Ltd. (Shanghai, China). Eudragit L100 (A kind of copolymer of methacrylic acid and methyl methacrylate (1:1)) was purchased from Evonik Degussa Co., Ltd. (Shanghai, Germany). Triethyl citrate (TEC) and dibutyl sebacate (DBS) were from Aladdin Reagents (Shanghai, China). Hydroxypropyl methylcellulose (HPMC), sodium starch glycolate and croscarmellose sodium were purchased from Hongyun Chemical Auxiliary Material Technology Co., Ltd. (Shanghai, China). Magnesium stearate and sodium lauryl sulphate (SLS) were purchased from Damao Chemical Reagent Factory (Tianjin, China). Carboxymethylcellulose sodium was purchased from Fuchen Chemical Reagent Factory (Tianjin, China). Other reagents used were of special reagent grade.

### 3.2. Preparation and Characterization of CCMS

In our previous reports, the CCMS were prepared by combining the ionic crosslinking and spray-drying method [24]. Dissolving CTS in acetic acid (1.0% *v*/*v*) produced a solution containing 1mg/mL CTS. Dissolving CAP in Tween-80 (0.5% *v*/*v*) produced a solution containing 1.5mg/ml CAP. Then the two solutions (CTS: CAP = 10: 1 *v*/*v*) were stirred with a magnetic stirrer for 15 min, and adjusted to pH 4.5 with sodium hydroxide. The 1% (*w*/*v*) polyphosphate sodium (TPP) was slowly added into the above-mentioned solution until a blue opalescence appeared. Then stirring was continued for 30 min. The morphology of CCMS was examined by scanning electron microscopy (Hitachi High Technologies S-3700N, Tokyo, Japan). The CCMS was found to be nearly spherical, and the external surfaces were almost smooth. The particle size and size distributions of CCMS were determined with particulate size description analyzer (Malvern Instruments Ltd Zetasizer^®^ 3000HS, Malvern, UK). The average particle size of CCMS was 4.5 μm. The encapsulation efficiency of CCMS was analyzed by High-performance liquid chromatograph (Waters e2695-2998, Milford, MS, USA). The encapsulation efficiency was 85.17%.

### 3.3. Preparation of CCMS Core Tablets

#### 3.3.1. Method for Preparation of CCMS Core Tablets

Core tablets of CCMS were prepared by wet granulation method [29]. CCMS and excipients were passed through no. 80 sieves. The CCMS was mixed with binder. The wet mass was forced through no. 24 sieves. The granules were dried to achieve a water content of 2% in an oven at 60 °C. The bulky cakes formed by the adhesion of particles and fine powder were removed from the dry granules by passing the material through no. 100 sieves and no. 24 sieves. The granules were mixed with lubricant and disintegrant. A 10 mm diameter circular die was used to prepare the core tablets (ZPW23 rotary tablet press, Tianxiang & Chentai Pharmaceutical Machinery Co., LTD, Shanghai, China).

#### 3.3.2. Formula Optimisation of CCMS Core Tablets

A single factor experiment was employed in the formula optimisation of CCMS core tablets. Nine formulae in Table 5 were designed to determine the optimal formula of the core tablet. The binders (carboxymethylcellulose sodium and HPMC) were investigated in formulae 1 and 2 firstly. Formulae 1, 3 and 4 were used to study the optimal concentration of carboxymethylcellulose sodium (3%, 5% and 7%). Then the lubricants (magnesium stearate and SLS) were investigated in formulae 3 and 5. The disintegrants (sodium starch glycolate and croscarmellose sodium) were investigated in formulae 6 and 7. Formulae 6, 8 and 9 were used to study the optimal dosage of sodium starch glycolate (16 mg, 24 mg and 32 mg). The hardness, disintegration time and appearance were variables to be investigated. To enable later consideration of the coating, we controlled the range of hardnesses between 50 and 60 N.

### *3.4.* Preparation of CCMS Enteric Coated Tablets

The enteric coating solution was prepared by dissolving Eudragit L100 and triethyl citrate in a 95% (*w*/*w*) aqueous ethanol solution for 12 h. Talc was added to the enteric coating solution and mixed well. The core tablets were wiped to remove fine powder and placed in a coating pan for preheating (inlet temperature 35–45 °C, 6–8 min). The coating pan was initially rotated slowly, and the speed was slowly increased to 25 rpm. The formal coating conditions used were as follows: inlet temperature, 40–45 °C; spray air pressure, 2–3 kg/cm^2^; pan rotation speed, 25 rpm. To prevent water infiltration and core tablet wear, hot air drying and spraying should not become continuous until the coat film is clear. The coated tablets were dried for 3 h at 45°C.

#### 3.4.1. Formulation Optimisation of Enteric Coating Solution

##### 3.4.1.1. Content of Talc

Talc can effectively prevent the adhesion phenomenon in the coating process, but too much talc readily precipitates and blocks the gun. Three levels of talc were investigated to identify the optimum talc content. These levels represented 10%, 25% and 40% of the polymer, respectively.

##### 3.4.1.2. Plasticisers

TEC and DBS were selected as plasticisers in these experiments. The dosage represented 20% of the Eudragit L100 and increased the coating weight by 4%. The release behaviour of the coating tablets in pH 6.8 phosphate buffer solution was monitored.

##### 3.4.1.3. Content of TEC

TEC was selected as the plasticiser, and the increase in coating weight was 4% relative to the fixed content of the other substances in the coating liquid. The dosage of TEC accounted for 10%, 20% or 30% of the Eudragit L100 in these experiments. The release behaviour of the coating tablets in pH 6.8 phosphate buffer solution was monitored.

##### 3.4.1.4. Coating Weight

It is known that the extent to which gastric acid erodes tablet coatings varies with the thickness of coat film covering the core tablet surface. Coating weight also has a significant impact on drug release. The coating weights of these tablets were 2%, 3% and 5%.

#### 3.4.2. Dissolution Research *in Vitro*

##### 3.4.2.1. Determination of Capsaicin Equilibrium Solubility

Several unsaturated capsaicin solutions and saturated capsaicin solution were prepared. The solvent was pH 6.8 phosphate buffer solution (0.20 M tribasic sodium phosphate: 0.1 N hydrochloric acid = 1:3). The temperature of the solutions was maintained at 37 ± 0.5 °C during the equilibrium solubility measurements using an incubation shaker (THZ-103B, Shanghai Yiheng Scientific Instrument Co. Ltd., Shanghai, China). Oscillation time was 48 h and oscillation frequency was 150 rmp. The sedimentation time was 24 h. Samples were filtered through 0.45 μm microfiltration membranes and injected into the HPLC under the chromatography conditions described above except column temperature was 37 °C.

##### 3.4.2.2. Dissolution Test of CCMS Core Tablet *in Vitro*

A USP dissolution basket apparatus (USP XXIII, 711) was employed to study the *in vitro* drug release of the CCMS core tablet. The experiment was conducted with 1000 mL phosphate buffer (pH 6.8, 0.20 M tribasic sodium phosphate and 0.1 N hydrochloric acid) at 100 rpm with the temperature controlled at 37 ± 0.5 °C for 13 h. At 0.5, 1, 2, 4, 7, 10, 13 h, samples were withdrawn through a syringe filter (pore size 0.45 μm) and replaced with an equal volume of fresh medium. The release behaviour of CAP from core tablets was monitored using an Intelligent Dissolution Tester (ZRS-8G, Tianjin Tianda Tianfa Technology Co., Ltd., Tianjin, China). Samples were then injected into the HPLC.

##### 3.4.2.3. Dissolution Research of CCMS Enteric Coated Tablet *in Vitro*

A USP dissolution basket apparatus (USP XXIII, 711) was employed to study the *in vitro* drug release from various batches. The experiment was conducted with 750 mL hydrochloric acid solution (0.1 N) at 100 rpm with the temperature controlled at 37 ± 0.5 °C for 2 h. At 0.5, 1, 2 h, samples were withdrawn through a syringe filter (pore size 0.45 μm) and replaced with an equal volume of fresh medium. After 2 hours, the baskets were immediately brought to the liquid level. The enteric-coating film was checked for ruptures and swelling behaviour in HCl. Then, tribasic sodium phosphate solution (250 mL, 0.20 M) was added to the assay solution for an additional 13 h, such that the experimental media became pH 6.8 phosphate buffer. At 2.75, 3, 3.5, 5, 7, 11 and 15 h, samples were withdrawn through a syringe filter (pore size 0.45 μm) and replaced with an equal volume of fresh medium. Samples were then injected into the HPLC. The release behaviour of CAP from enteric coated tablets was monitored using an Intelligent Dissolution Tester (ZRS-8G, Tianjin Tianda Tianfa Technology Co., Ltd., Tianjin, China). Samples were then injected into the HPLC.

#### 3.4.3. Release Kinetics

The capsaicin-chitosan microspheres are one of the matrix preparations which belonged to sustained-release preparations. There are several important kinetic models in the study of matrix drug release kinetics, such as Higuchi equation and Riter-Peppas equation [30]. And the First-order kinetics is a common drug release way of microspheres [31]. So, First-order, Higuchi and Riter-Peppas kinetic models were selected to fit the *in vitro* release data. The cumulative release (*Q*) of formulations optimized at different times (*t*) was calculated. The graph was plotted for cumulative percent of drug release *vs.* time. The *in vitro* release data of formulations optimized were fitted to the three equations with OriginPro 8.0 software (OriginLab, Northampton, MS, USA) to research the correlation coefficient “*R*” in three different release models. Different release model equations are as follows:

First-order Kinetic Equation:

(1) ln (1-0.01Q)=at

Higuchi Equation:

(2) $$Q=at^{1/2}+b$$

Ritger-peppas Equation:

(3) ln Q=a ln t+b

#### 3.4.4. HPLC Method

##### 3.4.4.1. Selection of Maximum Absorption Wavelength

CAP was dissolved in a moderate volume of methanol, diluted with pH 6.8 phosphate buffer and scanned over the 190–800 nm wavelength range. Capsaicin showed strong absorptions at 230 and 281 nm. Considering that CAP absorbed more strongly at 230 nm than at 281 nm, and because the excipients showed no interference, we chose 230 nm as the wavelength for CAP measurements.

##### 3.4.4.2. Chromatography Conditions

The column used was a Diamonsil C_18_ (5 μm, 250×4.6 mm, DIKMA, Beijing, China). The mobile phase was methanol: water (75:25), eluting at a flow rate of 1.0 mL/min. The detection wavelength used was 230 nm. The column was maintained at 30 °C. Injection volumes were 10 μL per sample.

##### 3.4.4.3. Specificity Investigation

Standard solutions of CAP, sample solutions of CAP and an enteric coated tablet without CAP solution, to be used as a negative reference, were prepared to investigate the specificity of the experiments and to determine peak area under the chromatography conditions described above.

##### 3.4.4.4. Linear Relationship Investigation

A precise amount of CAP standard substance (36.10 mg) was dissolved in methanol (50 mL) and diluted to 100 mL in a volumetric flask with pH 6.8 phosphate buffer (50 mL). This was taken as a reserve liquid. Standard solutions of 0.05, 0.10, 0.15, 0.30, 0.50, 0.80 mL were transferred to 10 mL volumetric flasks, diluted with pH 6.8 phosphate buffer and filtered through 0.45 μm microfiltration membranes. An aliquot of each filtrate (10 μL) was injected into the HPLC (Waters e2695-2998, Milford, MS, USA). Absorption spectra for each sample were taken under the chromatography conditions described above, and standard curves were drawn.

##### 3.4.4.5. Precision Experiment

We injected six separate 10 μL samples of the standard solution into the instrument, measured peak areas under the chromatography conditions described above and calculated relative standard deviations.

##### 3.4.4.6. Stability Experiment

An aliquot of the test solution was injected into the HPLC at 0, 4, 8, 16 and 24 h. Peak areas were measured under the chromatography conditions described above and relative standard deviations were calculated.

##### 3.4.4.7. Recovery Experiment

Five samples of CCMS powder (0.5 g each) were weighed precisely, added to 50 mL volumetric flasks and dissolved in methanol. The solutions were centrifuged for 15 min at 8000 rpm and filtered. Aliquots (1.5 mL) of the resulting filtrates were added to 25 mL volumetric flasks, treated with precise volumes of standard solution and further diluted to 25 mL with artificial intestinal juice. The final solutions were filtered through 0.45 μm microfiltration membranes.

### 3.5. Content Determination of CCMS Enteric Coated Tablets

Ten CCMS enteric coated tablets were stripped of their enteric coatings and pounded into fine powders. A sample (0.42 g) was collected from the fine powder and dissolved in methanol in a 100 mL volumetric flask. The solution was shaken well. The solution was centrifuged at 8000 rpm for 15 min and filtered. Samples (1 mL each) were withdrawn, transferred to 10 mL volumetric flasks and diluted with pH 6.8 phosphate buffer solution. The solutions were filtered through 0.45 μm microfiltration membranes and injected into the HPLC under the chromatography conditions described above.

## 4. Conclusions

The CCMS enteric coated tablets formulated with 25% talc, 20% TEC and 3% coating weight prevented release of the CAP in the pH 1.2 hydrochloric acid solution for a period of 2 h, and released in pH 6.8 phosphate buffer solution in a sustained manner. This property may reduce the irritation of the stomach significantly, minimize fluctuations of drug concentrations in blood and improve therapeutic performance. Optimized formulations showed a first-order drug release model in pH 6.8 phosphate buffer solution. The HPLC method used displayed excellent precision and accuracy. This research is the first report of the preparation and content determination of CCMS enteric coated tablets. All results reported above thus reflect that CCMS enteric coated tablets may be a potential drug delivery platform for sustained delivery of gastric irritant drugs.

## Figures and Tables

**Table 1.** Screening test results of formulation designation (means ± SD, *n* = 6).

Formulae	Hardness (*N*)	Disintegration time (min)	Appearance
1	55.2 ± 2.4	15.5 ± 1.5	+
2	57.2 ± 2.6	15.0 ± 0.9	−
3	58.4 ± 3.9	13.7 ± 0.5	+
4	56.4 ± 3.2	15.8 ± 1.3	+
5	55.0 ± 2.4	14.3 ± 1.2	+
6	54.6 ± 2.3	13.2 ± 0.8	+
7	56.2 ± 2.2	14.7 ± 1.0	+
8	53.8 ± 2.3	11.7 ± 0.5	+
9	54.8 ± 1.9	12.3 ± 0.8	+

Notes: +: Tablets are smooth and bright white with no phenomena of laminating, sticking and picking. −: Tablets are smooth, but dark yellow with some laminating, sticking and picking.

**Table 2.** Results of Recovery Experiments.

Known quantity (mg)	Adding quantity (mg)	Measured quantity (mg)	Recovery ratio (%)	Average recovery ratio (%)	RSD (%)
0.1923	0.1757	0.3673	99.60		
0.1928	0.1757	0.3685	100.00		
0.1955	0.1757	0.3725	100.74	99.42	1.15
0.1938	0.1757	0.3678	99.03		
0.1883	0.1757	0.3600	97.72		

Abbreviations: RSD, relative standard deviation.

**Table 3.** Release Kinetics of Formulation.

Drug release model	Kinetic equation	Mean relative percentage deviation	*R*^2^
First-order	ln (1 − 0.01*Q*) = −1.6295*t*	21.39%	0.99
Higuchi	*Q* = 8.3806*t*^1/2^ + 59.2705	6.00%	0.67
Ritger-peppas	ln*Q* = 0.1185 ln*t* + 4.1885	5.32%	0.74

Abbreviations: *Q*, cumulative percent of drug released; *t*, time; *R*, the correlation coefficient.

**Table 4.** Content of CAP (means ± SD, *n* = 10).

Batch	Content of CAP (mg/each tablet)	Mean Content (mg/each tablet)
1207014	5.45 ± 0.01	
1207015	5.44 ± 0.02	5.44
1207016	5.43 ± 0.01	

Abbreviations: CAP, capsaicin.

**Table 5.** Formulae Designation of CCMS Core Tablets.

Excipients		Formulae								

		1	2	3	4	5	6	7	8	9
Drug	CCMS (mg)	400	400	400	400	400	400	400	400	400

Binders	Carboxymethylcellulose Sodium (Right amount, *w*/*v*)	5%		3%	7%	3%	3%	3%	3%	3%
	HPMC (Right amount, *w*/*v*)	-	5%	-	-	-	-	-	-	-

Lubricants	Magnesium Stearate (mg)	4	4	4	4	-	4	4	4	4
	SLS (mg)	-	-	-	-	4	-	-	-	-

Disintegrants	Sodium Starch Glycolate (mg)	-	-	-	-	-	24	-	16	32
	Croscarmellose Sodium (mg)	-	-	-	-	-	-	24	-	-

Notes: Percentage is expressing concentration in tablet. Abbreviations: CCMS, capsaicin-chitosan microspheres; HPMC, hydroxypropyl methylcellulose.
